# A three-wave longitudinal study on the relation between commuting strain and somatic symptoms in university students: exploring the role of learning-family conflicts

**DOI:** 10.1186/s40359-021-00702-7

**Published:** 2021-12-20

**Authors:** Mathias Diebig, Jian Li, Boris Forthmann, Jan Schmidtke, Thomas Muth, Peter Angerer

**Affiliations:** 1 Institute of Occupational, Social and Environmental Medicine, Heinrich Heine University Düsseldorf, Universitätsstr. 1, 40225 Düsseldorf, Germany; 2 Department of Environmental Health Sciences, Fielding School of Public Health, School of Nursing, University of California, Los Angeles, Los Angeles, USA; 3 Institute of Psychology in Education, University of Münster, Münster, Germany

**Keywords:** Commuting strain, Medical students, Multilevel research, Somatic complaints

## Abstract

**Background:**

We examine the role of learning-family conflicts for the relation between commuting strain and health in a sample of medical university students. The first goal of the study was to investigate the mediating role of learning-family conflicts. The second goal was to extend the temporal view on relations between study variables. Therefore, we differentiated long-term systematic change among variables over a period of two-years from a dynamic perspective with repeated commuting events on the individual level of analyses.

**Methods:**

We applied a multilevel research design and collected survey data from 128 medical students on three points in time (*N* = 339 measurement points). Participants informed about commuting strain, learning-family conflicts, somatic symptoms, as well as commuting distance and time.

**Results:**

Bayesian multilevel analyses showed that results differed with regard to level of analysis: while learning-family conflicts mediated the relation between commuting strain and somatic symptoms on a systematic aggregation-level perspective of analysis (indirect effect estimate_between_ = 0.13, *SE* = .05, 95% CI [0.05; ∞), Evidence Ratio = 250.57), this was not the case on the dynamic event perspective (indirect effect estimate_within_ = 0.00, *SE* = 0.00, 95% CI [− 0.01; ∞), Evidence Ratio = 0.84).

**Conclusions:**

We demonstrated that learning-family conflicts explain why commuting may have unfavorable effects on health for medical students. We also showed that it is the long-term commuting experience that is related to health complaints and not the single commuting event. This means that short-term deviations from general levels of commuting strain do not cause somatic symptoms, but general high levels of commuting strain do instead.

## Background

Commuting between university and home describes a daily routine for most university students. In a survey with 67.007 students from 248 universities in Germany, 29% of these students did not live in the same city as their university and, therefore, had to commute [1]. Results of this survey show that the way between home and university takes 33 min on average. Twenty-one percent of the students even have to commute more than 45 min.

Evidence from previous research has suggested that daily commuting is associated with commuters’ physical health as well as psychological well-being in several ways [2]. Individuals who have to commute between work and home report more health problems than non-commuting controls. In particular, they report more psychosomatic complaints, reduced well-being, and greater general dissatisfaction with life [3–7]. With regard to physical health complaints, research has demonstrated that daily commuters report suffering from headache and neck pain [3] as well as an increased blood pressure [5]. However, these findings were exclusively derived from working populations not considering the group of students. Students represent a considerable proportion of commuters who are confronted with the burden of daily commuting in addition to the burden of studying [8]. For instance, medical students are exposed to high demands which have been linked to negative health outcomes, such as depressive symptoms [9], musculoskeletal complaints [10], and reduced quality of life [11]. Therefore, it is important to explore demands associated with commuting that medical students face causing severe health problems. In this study, we test which role commuting plays as one possible demand of medical students and, also, explore unfavorable consequences of commuting in this particular population.

Previous research has mainly focused on effects of travel impedance (e.g. all stressors of commuting that impede one’s commute, such as traffic congestion) on various aspects of health [12]. In this case, travel impedance is characterized as a function of commuting distance and time, thus representing differences between longer or shorter commutes. Although most previous studies have demonstrated that long commuting times are associated with impaired health of commuters [13–16], recent research has shown that particularly longer commutes have not been associated with low life satisfaction in all cases [17]. Researchers have assumed that these commuters had been successful in balancing the negative aspects of commuting against other benefits such as better access to employment, earnings or housing. What is missing in this line of research is an explanation *why* particularly time is a crucial factor of commuting that drives effects. We complement existing research by focusing on the role of learning-family conflicts (*LFC*) to explain why commuting is associated with poor health [8, 18]. LFC is closely linked to the aspect of commuting time since time spent on the commute is interfering with potential family time; thus, causing potential conflicts between the domain family and university. LFC may function as a generating mechanism to describe why commuting causes health problems. We, therefore, test whether commuting results in an incompatibility between learning arrangements and the family domain that ultimately leads to somatic symptoms. Initial research on LFC and commuting suggests that the time demands accompanying long commutes are important to explain its relation to LFC and stress [19, 20]. Further, meta-analytical evidence shows that LFC is related to health problems as well as somatic symptoms [21]. As LFC has been previously linked to commuting as well as health problems, we expect LFC to mediate the relation between commuting strain and somatic symptoms in our study.

In addition, we aim to adopt a transactional understanding of stress to show that it is not only the mere presence of a stressor that causes somatic symptoms, but also the personal interpretation about how to cope with this stressor. Transactional stress models assume that individuals experience stress when a situation is relevant (primary appraisal) and individuals lack resources to overcome this situation (secondary appraisal; [22]). This view on stress proposes that individuals evaluate whether a potential demanding situation may be solved or not. Thus, the personal background of commuting individuals may play a central role when assessing health-effects as the presence of a stressor—i.e., threat of arriving late—might be evaluated differently across student commuters [23]. In accordance, previous research has shown that the relation between commuting time and health-related quality of life was mediated by perceived stress [24], which may function as an appraisal in terms of the transactional stress model. We test this assumption in a pilot study by focusing on individual’s perceived commuting strain as a function of commuting distance and time to operationalize a transactional stress perspective on commuting.

### A time-sensitive perspective on commuting strain

A second way in which our research complements existing literature is by extending the temporal view on relations between study variables. An important methodological aspect of research in the commuting-health interface is the situational dependency of the commute as well as of the individuals’ response to it. It is reasonable to assume that aspects of commuting fluctuate over time because each commute might be affected by different external events and, therefore, poses different challenges for commuters. Only very few studies have attempted to account for these aspects. In a study with 45 bus commuters, daily data over a period of 15 workdays showed that the amount of commuting stressors in the morning influenced commuters’ morning commuting strain [25]. This morning commuting strain, in turn, had a negative impact on commuters’ self-regulation at work and was exacerbated by daily family interference with work in the morning.

By implementing a multilevel research design and measuring study variables on three different time points in our study, we may study relations from a dynamic event perspective to understand the relation between commuting and health as a function of different events for each individual (within-person level). However, we also extended this perspective by adding the notion that commuting effects may also change over the course of two years and display systematic change patterns from a long-term perspective (between-person level). Previous research has mainly focused on between-person relations or within-person relations in isolation [26]. However, we assume that time plays a crucial role when explaining relations between commuting and health. Previous work has demonstrated that the impact of certain demands on health may function contrarily at the between-person and the within-person level of analyses [27]. It is, therefore, necessary to systematically investigate if relationships between demands and health are similar or differ across levels of analysis to answer whether an aggregate construct at the between-person level is more than the sum of its lower level counterpart at the within-person level [28]. Therefore, the purpose of our study was to enable a better understanding between long-term change patterns and dynamic re-occurring events in commuting-health research by considering relations on different levels of analysis while exploring the role of LFC for the commuting-health interface. With this, we are able to systematically test whether relationships between constructs are similar or different across levels of analysis [28, 29] and to generate knowledge whether the aggregated commuting experiences on the long run is more than the sum of its lower level events at the individual level.

### Aims of the present study

We examine the role of learning-family conflicts for the relation between commuting strain and health in a sample of medical university students. The first goal of the study was to investigate the mediating role of learning-family conflicts for the relation between commuting strain and health to gain a better understanding on relevant demands of medical university students’ lives and their potential consequences on health. The second goal was to extend the temporal view on relations between study variables and to consider long-term systematic change among variables over a period of two-years in comparison with dynamic repeated events on the individual level of analyses.

### Pilot study

We conducted a pilot study to test whether commuting strain is a suitable indicator of the subjective commuting experience. This was tested by linking commuting strain to commuting distance as well as time as these two aspects of commuting have been shown to be main drivers of an individual’s reaction to commuting [4, 5, 12]. We used data from a survey across medical students at an university in Germany to test for associations between commuting distance and time with commuting strain.

#### Sample and procedure

In total 548 students of a German university took part in our pilot study on two measurement points with a two-year follow-up. Of them, 70% were female and mean age of participants was 23.35 years (*SD* = 2.96). Age ranged from 19 to 39 years. Students were in the third year of study on measurement point one and in the fifth on measurement point two. Of the students, 27% commuted by foot/bike to university, 20% by public transport, and 13% by car. Some students also used a combination of different commuting means to get to university. By foot/bike and public transport 22%, by public transport and car 15%, and by car and foot/bike 3%.

#### Measures

We measured *commuting strain* with one single-item “How strongly do you feel stressed by long journeys to university campus?”. The response format ranged from 0 (*not at all*), 1 (*somewhat*), 2 (*strongly*), to 3 (*very strongly*). We developed our measure of commuting strain and used the pilot study to validate this single-item scale. Also, we asked for the distance between home and university in kilometers (*commuting distance*) as well as the amount of time it takes to get to university from home (*commuting time*).

#### Controls

We controlled for both sex and age of measurement point 1 in the regression analysis. In addition, as we collected data on two different time points with a two-year interval, we calculated a difference score for commuting distance as well as time (T_1_ − T_2_). A difference of zero means that students did not change their living location. A difference greater zero means that students moved nearer to university and, therefore, needed less time to commute. Whereas a difference score smaller than zero means that students moved farther away and needed more time to get to university. We controlled for both difference scores and used commuting distance and time of t_1_ as predictors in our regression model, sex and age as control variables, and commuting strain of t_2_ as dependent variable.

#### Results

On average, students lived 12.79 km (*SD* = 17.36, *Min* = 0, *Max* = 100) away from university and commuted for about 27.56 min (*SD* = 25.51, *Min* = 0, *Max* = 180; cf. Table 1). We grouped answers of students to the three categories no relocation, relocation nearer to university, and relocation farther away from university. Most students did not move (57%), 28% moved nearer to university, and 11% moved farther away.

**Table 1:** Means (M), standard deviations (SD), and correlations of pilot study variables

	*M*	*SD*	1	2	3	4	5	6
1. Age	23.35	2.96	–					
2. Sex^1^	0.71	0.46	− .15**	–				
3. Diff distance^2^	1.66	16.52	.01	− .03	–			
4. Diff time^2^	3.89	23.75	− .04	.05	.68**	–		
5. Commuting distance	12.79	17.36	.15**	.01	.50**	.36**	–	
6. Commuting time	27.56	25.51	.13**	.10*	.34**	.59**	.75**	–
7. Commuting strain	1.27	1.08	.12**	.08	− .13**	− .13**	.47**	.46**

*N* = 548

^1^sex coded as 1 = female and 0 = male

^2^Difference scores for commuting distance and time calculated as values of t_1_ minus values of t_2_ of the respective variable

^*^p < .05; **p < .01

Multiple regression analysis was used to test if control variables (age, sex, difference score distance, and difference score time) as well as main study variables (commuting distance and commuting time) predicted participants' ratings of commuting strain (cf. Table 2). We inserted age, sex, commuting distance and commuting time from measurement point 1 in the regression, commuting strain from measurement point 2, and the difference scores as change score T_1_ − T_2_. The results of the regression indicated the six predictors explained 49% of the variance (*R*^2^ = 0.49; *F*(6,507) = 82.02, *p* < 0.01). Multicollinearity was not a concern as collinearity statistics for all predictor variables were in an acceptable range (Tolerance indices for all predictors > 0.15 and VIF < 6.00). It was found that control variables sex (*β* = 0.02, *SE* = 0.08, *ns*) and age (*β* = 0.01, *SE* = 0.01, *ns*) were not related to commuting strain. Both difference scores were significant predictors for commuting strain (difference score distance: *β* = − 0.26, *SE* = 0.00, *p* < 0.01; difference score time: *β* = − 0.39, *SE* = 0.00, *p* < 0.01), in the way that the nearer (farther away) students moved to university, the less (more) commuting strain they experienced. In addition, results showed that commuting distance (*β* = 0.37, *SE* = 0.00, *p* < 0.01) as well as commuting time significantly predicted commuting strain (*β* = 0.47, *SE* = 0.00, *p* < 0.01). In sum, findings show that perceived commuting strain is a suitable indicator of the subjective commuting experience since it is strongly related to the objective parameters of commuting distance and time.

**Table 2:** Results of regression analyses of pilot study variables

	Commuting strain		
	*b*	*SE*	*β*
*Controls*			
Age	.00	.01	.01
Sex^1^	.06	.08	.02
Diff distance^2^	− .02	.00	− .26**
Diff time^2^	− .02	.00	− .39**
*Study variables*			
Commuting distance	.02	.00	.37**
Commuting time	.02	.00	.49**
*R*^*2*^	.49**		
*F*	82.02		

*N* = 548

^1^sex coded as 1 = female and 0 = male

^2^Difference scores for commuting distance and time calculated as values of t_1_ minus values of t_2_ of the respective variable

^*^p < .05; **p < .01

## Methods

### Procedure

In the main study, we used data from an ongoing prospective cohort study that started in 2012 among medical students of a German university. Two cohorts are included in the study. The first cohort started their medical training in October 2012 and the second cohort in October 2013. Students were invited to take part in the study at the beginning of their first semester of medical training. At this point in time, we provided one initial survey to the students. In this initial survey, we asked participants to inform about age and sex, as well as about their living and commuting environment. In our main survey, participants gave information about commuting strain, LFC as well as somatic symptoms. This survey was distributed to participants on three measurement occasions (at the beginning of semesters 5, 7, and 9 with a time lag of 1 year each). Data of the different time points of the participants were matched by a self-generated code.

The universities’ institutional ethics committee approved the study. All procedures contributing to this work comply with the ethical standards of the relevant national and institutional committees on human experimentation and with the Helsinki Declaration of 2013. All participants submitted written informed consents.

### Sample

One hundred and twenty-eight medical students took part in our study and gave information about study variables (commuting strain, LFC, and somatic symptoms) on three different points in time (*N* = 339 measurement points). Forty-five students completed the main survey on two measurement points and 83 completed the main survey on all three measurement points. Mean age of the sample was 20.70 years (*SD* = 3.47) and 70% of the students were female.

### Measures

All instruments were presented in German language.

#### Commuting strain

We measured commuting strain with one single item “How strongly do you feel stressed by long journeys to university campus?” according to the description of the scale in the pilot study. The response format ranged from 1 (*not at all*), 2 (*somewhat*), 3 (*strongly*), to 4 (*very strongly*).

#### Learning-family conflicts

To measure LFC, we used five items from the Copenhagen Psychosocial Questionnaire (COPSOQ; [30]) and adapted items to the learning context [18]. A sample item is “The demands of my learning interfere with my home and family life.” Items were answered on a 5-point scale ranging from 1 (*strongly disagree*) to 5 (*strongly agree*). Therefore, a high score represents high LFC. Cronbach’s alpha was calculated as mean internal consistency averaged over all measurement points with average α = 0.93. This value corresponds to the alpha value reported in the initial validation study of the German version of the COPSOQ [31].

#### Somatic symptoms

We used the Patient Health Questionnaire (PHQ-15; [32]) to screen for the severity of somatic symptoms. The PHQ-15 comprises 15 somatic symptoms from the PHQ (e.g., sleep problems, backpain, headache, heart pound or race). Participants were asked to describe whether one of the symptoms had bothered them during the past four weeks. Answers ranged from 1 (*not bothered at all*), 2 (*bothered a little*), to 3 (*bothered a lot*). The average Cronbach’s alpha value over all measurement points was α = 0.77 and was comparable to coefficients reported in other studies using the PHQ-15 in German language [33].

#### Controls

Control variables were used to rule out alternative explanations for our results. We controlled for age (in years) and sex as well as commuting distance and time with all control variables collected on measurement point 1.

### Analytical procedure

The data for the present study was hierarchical in nature with measurement points (Level 1) nested within individuals (Level 2). We applied Bayesian multilevel analyses to test the research model with the package brms [34, 35] for the statistical software R [36]. Main study variables were all collected on Level 1. Control variables were measured at the individual level on Level 2. Therefore, our model represents a 1–1–1 mediation model. We tested for multilevel mediation using multilevel structural equation modeling (MSEM) applying the procedure by Preacher and colleagues [37, 38] that was implemented to within-person research by Hülsheger et al. [28].

However, we had to adapt the approach to the data in the current study. In particular, the main predictor commuting strain was measured as an ordinal variable. Consequently, this was taken into account by monotonic effect modeling for commuting strain [39]. Monotonic effect modeling provides an estimate of the average increase of the dependent variable when the ordinal predictor is increased from any of the ordered categories to the adjacent category. Thus, this approach preserves the common interpretation of linear regression coefficients. In addition, this approach provides a set of simplex parameters that indicate the expected difference between two adjacent categories as a proportion of the difference between the lowest and the highest category (see 39). In other words, the simplex parameters indicate for which change between adjacent categories the expected change in the dependent variable would be highest.

For monotonic effect modeling at the within and between person level, the person-centering approach from common multilevel modeling approaches for continuous variables had to be mimicked for commuting strain. To preserve the properties of an ordinal variable at both levels, we used the 50% quantile according to the definition provided by Hyndman and Fan [40] as an aggregation statistic at the person level. Importantly, this statistic preserves the same four ordered categories as the original item to measure commuting strain. Then, commuting strain was centered around this person level aggregate score. These deviations resulted in integers reflecting commuting strain as an ordinal within-level variable.

The amount of missing data was 8%. Thus, we checked the pattern of missingness. The two-step procedure suggested by Jamshidian and Jalal [41] as implemented in the R package MissMech [42] revealed that the assumption of missing completely at random (MCAR) could not be refuted for the data (Hawkin's Test: *p* = 0.002, non-parametric test of homoscedasticity: *p* = 0.566; tested with alpha = 0.20 to be conservative when retaining the null hypothesis is of interest). Then, we simply omitted rows with missing values only on all main study variables at the first time point (16 cases), at the second time point (7 cases), and the third time point (18 cases). Missing values beyond these patterns were imputed by multiple imputation as implemented by the R package mice [43]. For better imputation results, we used a few other variables from the larger project that were correlated with control variables requiring imputation. These additional variables were place of living (parents’ home, own home, or dormitory), general self-efficacy [44], personal attitude of the importance of completing one’s studies (1 = not important to 4 = very important), and empathy [45]. The final data analysis was based on ten imputed datasets which were combined into final results in a straight forward manner by the brms function brm_multiple(). This treatment of missing values was justified by the result that the MCAR assumption could not be refuted.

To conclude, the outlined procedure enables us to compare results on both levels of analysis. On the one hand, results display the role of a person’s level of commuting strain on somatic symptoms (Level 2: between-person). On the other hand, results inform on the role of a person’s variations from his or her own level of commuting strain for somatic symptoms (Level 1: within-person).

## Results

Table 3 reports the descriptive statistics, reliabilities, and correlations among all study variables. We calculated the between-person variance for somatic symptoms to justify multi-level analyses. ICC(1) for somatic symptoms was 68% indicating that substantial amounts of variance remain at the within-person level. The same applied for LFC (ICC(1) = 66%). For the ordinal variable commuting strain, Krippendorff’s α [46] was calculated as a measure of reliability of between-person variation. The found estimate of 0.55 was again indicative for variation in commuting strain at both levels of analysis (Table 3 includes also the ICC(1) for commuting strain which was calculated for conventional reasons and did not yield a different result).

**Table 3:** Means, standard deviations, reliabilities, and correlations of between- and within-level variables

	*M*	*SD*	ICC	1	2	3
*Between-level*						
1. Age	20.70	3.47	–	–		
2. Sex^1^	0.30	0.46	–	.01	–	
3. Commuting distance	25.48	28.47	–	− .06	.04	–
4. Commuting time	46.05	35.02	–	− .05	− .05	.80**
*Within-level*						
1. Commuting strain	1.92	1.02	.60^2^	–		
2. Learning-family conflict	3.46	1.11	.66	− .01	(.93)	
3. Somatic symptoms	1.47	0.29	.68	− .02	.04	(.77)

*N*_*Within*_ = 339; *N*_*Between*_ = 128. Cronbach’s alphas on the diagonal for day-level variables are mean internal consistencies averaged over all measurement points

^1^sex coded as 1 = female and 0 = male

^2^ For commuting strain ICC was calculated for conventional reporting, but caution is required because of the ordinal scale of this variable

^**^ p < .01; * p < *.05*

First, convergence of the chains by the Rhat statistic was checked and was found to be satisfactory with a maximum value of 1.01 (all Rhat values should be < 1.10) across all model parameters. The results of multilevel regression analyses are reported in Table 4. At the between-person level, commuting strain was substantially related to LFC (i.e., the 95% credible interval for regression coefficient *b* did not cover zero) while controlling for sex, age, commuting distance and time. The estimate indicates that on average an increase from one ordinal category to the next higher category would result in an expected increase of the dependent variable by *b* = 0.85. The simplex estimates further revealed that the steps from *not at all* to *somewhat*, and from *somewhat* to *strongly* yielded equal expected changes in LFC, whereas the step from *strongly* to *very strongly* resulted in a higher expected change of LFC.

**Table 4:** Results of Bayesian multilevel regression analyses

	Mediator: Learning-family conflict			Outcome: Somatic symptoms		
	*Est*	l-95% CI	u-95% CI	*Est*	l-95% CI	u-95% CI
*Within-level*						
Intercept	0.02	− 0.21	0.30	0.01	− 0.06	0.07
Commuting strain^1^						
*b*	− 0.05	− 0.52	0.34	− 0.03	− 0.17	0.09
simo[1]	0.28	0.01	0.74	0.25	0.01	0.72
simo[2]	0.23	0.01	0.67	0.20	0.01	0.65
simo[3]	0.21	0.01	0.63	0.20	0.01	0.63
simo[4]	0.29	0.01	0.75	0.35	0.01	0.82
Learning-family conflict				0.01	− 0.02	0.04
*Between-level*						
Intercept	2.14	1.16	3.12	1.18	0.95	1.41
Age	0.06	0.02	0.11	− 0.01	− 0.02	− 0.00
Sex^2^	− 0.53	− 0.88	− 0.18	− 0.12	− 0.20	− 0.04
Commuting distance	0.06	− 0.22	0.33	0.03	− 0.02	0.09
Commuting time	− 0.07	− 0.35	0.22	− 0.05	− 0.11	0.01
Commuting strain						
*b*	0.85	0.21	1.49	0.13	0.02	0.24
simo[1]	0.20	0.01	0.58	0.49	0.07	0.89
simo[2]	0.20	0.01	0.65	0.27	0.01	0.72
simo[3]	0.60	0.10	0.92	0.24	0.01	0.68
Learning-family conflict				0.16	0.12	0.20
*R*^2^ *within*^3^	.00	.00	.02	.01	.00	.03
*R*^2^ *between*^3^	.19	.09	.30	.48	.39	.56

*N*_*Within*_ = 339; *N*_*Between*_ = 128. Est. = Estimate; l-95% CI = lower bound of a 95% credible interval; u-95% CI = upper bound of a 95% credible interval

^1^The b coefficient can be interpreted as the average change in the dependent variable when the ordinal predictor commuting strain increases from one category to the next higher category (analogous to the common interpretation of regression coefficients; see Bürkner & Charpentier, 2018). ^2^Sex coded as 1 = female and 0 = male. ^3^Bayesian *R*^2^ as proposed by 55 [55]

Also, commuting strain predicted somatic symptoms on the between-level of analysis. On average an increase from one ordinal category to the next higher category would result in an expected increase of the somatic symptoms by *b* = 0.13. The simplex estimates further revealed that the step from *not at all* to *somewhat* resulted in the highest expected change of somatic symptoms, whereas all other simplex parameters were comparable in size. In addition, somatic symptoms were predicted by LFC implying a mediation. The posterior probability for the hypothesis that the indirect effect is > 0 (vs. < 0) on the between-level was 1.00 (Estimate_between_ = 0.13, *SE* = 0.05, 95% *CI* [0.05; ∞), Evidence Ratio = 250.57), showing strong evidence that the relation between commuting strain and somatic symptoms was mediated by LFC.

Contrarily, on the within-person level, none of the tested direct relations (monotonic effects in case of commuting strain) was substantial. Also, the indirect effect of commuting strain on somatic symptoms via LFC was zero (Estimate_within_ = 0.00, *SE* = 0.00, 95% *CI* [− 0.01; ∞), Evidence Ratio = 0.84).

## Discussion

The aim of the present study was to explore the role of LFC for the commuting-health relationship. Further, we wanted to extend the temporal view on relations between commuting and health by differentiating long-term systematic change among variables over a period of two-years from short-term consequences of dynamic repeated commuting events on the individual level of analyses.

Results showed that it is the long-term commuting experience that is related to poor health and not the single commuting event. This means that short-term deviations from general levels of commuting strain did not cause somatic symptoms in our study. E.g. if someone feels sometimes more and sometimes less stressed by a commute has a smaller effect on health than the average long-term commuting strain over the two-year time frame. These results highlight that it is important to look at relations from a temporal, multilevel perspective [26, 47]. Particularly for the commuting research this is a noteworthy finding as commuting events are highly depending on situational factors like high traffic congestion or train cancellation which have a high variability i.e., may vary from one day to another [25, 48, 49]. Our results resemble results of previous work that has reported longitudinal evidence of adverse associations between commuting distance and physical inactivity, overweight, and disturbed sleep [14].

In addition, we showed that the mechanism to explain the relation between commuting strain and somatic symptoms was determined by LFC. As recent research has shown that health-effects of commuting may depend on personal aspects of commuters (e.g., individual work arrangements; 15), it is important to further explore the role of third variables in this area of research. In line with findings of previous studies that showed a positive relation between commuting time and learning-family conflict [19], we demonstrated the role of LFC for the commuting-health interface. However, our findings show that it is rather the aspect of commuting strain that causes LFC instead of the factor of commuting time. Although previous studies have identified commuting time as one important driver of adverse health effects of commuting [7, 15, 16, 50], these studies do not present clear evidence for explaining the mechanisms through which commuting time enfolds its effects. As we demonstrate that commuting strain is related to poor health—while controlling for commuting distance and time—findings encourage us to rethink current explanations for the commuting health-interface that solely focus on duration and length of the commute. While in our study neither commuting distance nor time were related to somatic symptoms, it seems to be the individual’s subjective stress experience that drives relations between commuting and health [24]. Therefore, our results suggest a transactional viewpoint on the commuting stress reaction in which the appraisal of a stressful situation and not the mere presence of a stressor causes strain [22].

Finally, we demonstrated that LFC mediated the relation between commuting strain and somatic symptoms in a sample of medical university students. We showed that commuting is linked to students’ health status and, therefore, represents one central demand of medical studies. This gets even more important as course presence has been shown to be highly relevant for medical students. Despite exceptional circumstances during the COVID-19 pandemic, students still need to be present at university courses because of the inherent characteristics of their curricula [51]. Therefore, to be successful as a medical student, one should regularly physically attend courses at the university building [51]. That is, there is a need for these students to get to university. If now students do neither live nearby the university building nor in the same city, they have to commute.

### Limitations and future directions

Although our study is characterized by certain strengths i.e., longitudinal data collection over the course of two years and advanced application of multilevel modelling, there are inherent limitations associated with the design of our study. We could only collect information on participants living as well as commuting situation in our initial survey. Therefore, we do not know whether commuting situation changed over time, because participants moved. Yet, pilot study results showed that distance and time of the commute had a significant effect on commuting strain while controlling for potential changes of the living situation. In addition, as we mainly focus on the influence of commuting strain on health, the potential confounding effect of participants’ relocation is negligibly.

Our measure of commuting strain relied on a single item that represented a general retrospective self-report rating of commuting strain. Research from the field of travel satisfaction has shown that this kind of measure might be biased by forgetting, inaccuracy, or stereotypical beliefs [49]. As a result, commuters report general perceptions of the commute that may differ from their actual commuting experience [52, 53]. Therefore, future research should rely on measuring commuting strain during the commute to avoid memory distortions. Therefore, the aim of future research should also be to develop suitable measures to assess commuting strain unobtrusively during the commute.

Also, while exploring the role of LFC to explain effects of commuting on health, we did not differentiate between the two directions of LFC e.g., if learning interferes with the family role or if the family interferes with learning. Since both directions might be related to commuting independently [21], future research should consider the family background of participants to make conclusions on this topic in detail.

Lastly, we administered our data collection at only one medical faculty in Germany. Therefore, the representativeness of our sample and the generalizability of findings are limited. Yet, the use of rather homogeneous samples may help to more easily identify and quantify effects within a new research phenomenon by holding potential confounding factors constant while reducing irrelevant variance [54].

## Conclusion

Our findings reveal that it is predominantly a person’s general aggregation-level of commuting strain over time that explains whether that person suffers from somatic symptoms. This means that deviations from general levels of commuting strain do not cause somatic symptoms but general high levels of commuting strain do instead. Moreover, learning-family conflicts play an important role in explaining why individuals experience somatic symptoms as a result of commuting above and beyond the objective parameters of commuting distance and time.

## Data Availability

The data of the current study are available upon reasonable request from the corresponding author.

## Abbreviations

COPSOQ: Copenhagen Psychosocial Questionnaire
LFC: Learning-family conflicts
MCAR: Missing completely at random
MSEM: Multilevel structural equation modeling
PHQ: Patient Health Questionnaire
